# Supplementary material for: Anorectal Remodeling in the Transitional Zone with Increased Expression of LGR5, SOX9, SOX2, and Keratin 13 and 5 in a Dextran Sodium Sulfate-Induced Mouse Model of Ulcerative Colitis
Source: Int J Mol Sci. 2024 Nov 26;25(23):12706. doi: 10.3390/ijms252312706 (PMC11640979; doi:10.3390/ijms252312706)
Supplement: Supplementary file 1 [file ijms-25-12706-s001.zip › ijms-3322316-supplementary.pdf]

Supplemental Information

Anorectal remodeling in the transitional zone with increased expression of LGR5, SOX9, SOX2, and keratin 13 and 5 in a dextran sodium sulfate-induced mouse model of ulcerative colitis

Mio Kobayashi[1,2], Tatsuya Usui[3], Mohamed Elbadawy[3,4], Tetsuhito Kigata[5], Masahiro Kaneda[5], Tomoaki Murakami[6], Takuma Kozono[7], Yoshiyuki Itoh[7], Makoto Shibutani[1], Toshinori Yoshida[1]

1. Laboratory of Veterinary Pathology, Cooperative Department of Veterinary Medicine, Tokyo University of Agriculture and Technology, 3-5-8 Saiwai-cho, Fuchu-shi, Tokyo 183-8509, Japan
2. Cooperative Division of Veterinary Sciences, Tokyo University of Agriculture and Technology, 3-5-8 Saiwai-cho, Fuchu-shi, Tokyo 183-8509, Japan
3. Laboratory of Veterinary Pharmacology, Cooperative Department of Veterinary Medicine, Tokyo University of Agriculture and Technology, 3-5-8 Saiwai-cho, Fuchu-shi, Tokyo 183-8509, Japan
4. Department of Pharmacology, Faculty of Veterinary Medicine, Benha University, Moshtohor, Toukh 13736, Elqaliobiya, Egypt
5. Laboratory of Veterinary Anatomy, Cooperative Department of Veterinary Medicine, Tokyo University of Agriculture and Technology, 3-5-8 Saiwai-cho, Fuchu-shi, Tokyo 183-8509, Japan
6. Laboratory of Veterinary Toxicology, Cooperative Department of Veterinary Medicine, Tokyo University of Agriculture and Technology, 3-5-8 Saiwai-cho, Fuchu-shi, Tokyo 183-8509, Japan
7. Smart-Core-Facility Promotion Organization, 3-5-8 Saiwai-cho, Fuchu-shi, Tokyo 183-8509, Japan

Corresponding author:
Toshinori Yoshida, DVM, PhD, DJCVP, DJSTP
Laboratory of Veterinary Pathology, Cooperative Department of Veterinary Medicine, Tokyo University of Agriculture and Technology, 3-5-8 Saiwai-cho, Fuchu-shi, Tokyo 183-8509, Japan
E-mail: yoshida7@cc.tuat.ac.jp
Tel : +81-42-367-5874
Fax : +81-42-367-5771

Table S1. Antibody lists for immunohistochemistry.

Antigen	Host	Clonality	Clone name	Dilution	Antigen retrieval	Manufacture
E-cadherin	Mouse	Monoclonal	M168	1:500	Autoclaving pH6[a]	Abcam (Cambridge, UK)
β-catenin	Rabbit	Polyclonal	n.a.	1:250	Microwaving pH6[c]	Santa Cruz Biotechnology, Inc. (Dallas, Texas, USA)
Ki-67	Rabbit	Monoclonal	MIB-1	1:500	Autoclaving pH6[a]	Dako (Glostrup, Denmark)
Tumor protein p63 (p63)	Rabbit	Polyclonal	n.a.	1:1000	Autoclaving pH6[a]	Proteintech (Illinois, USA)
5-Bromo-2'-deoxyuridine (BrdU)	Rat	Monoclonal	BU1/75(ICR1)	1:800	Autoclaving pH6[a]	Abcam (Cambridge, UK)
Sex determing region on Y-box transcription factor 2 (SOX2)	Mouse	Monoclonal	9-9-3	1:4000	None	Abcam (Cambridge, UK)
Leucine-rich repeat-containing G-protein-coupled receptor 5 (LGR5)	Mouse	Monoclonal	n.a.	1:400	Autoclaving pH6[a]	Abcam (Cambridge, UK)
Sex determing region on Y-box transcription factor 9 (SOX9)	Rabbit	Monoclonal	n.a	1:1000	Autoclaving pH6[a]	Abcam (Cambridge, UK)
Phosphorylation of histone H2AX at serine 139 (γ-H2AX)	Mouse	Monoclonal	3F2	1:1000	Autoclaving pH6[a]	Abcam (Cambridge, UK)

Abbreviation: n.a, not applicable.

a. Autoclaved at 121°C for 10 min in 10 mM citrate buffer (pH 6.0).

b. Microwaved at 90°C for 10 min in Dako target retrieval solution (pH 9.0).

c. Microwaved at 90°C for 10 min in 10 mM citrate buffer (pH 6.0).

d. Autoclaved at 120°C for 10 min in Dako target retrieval solution (pH 9.0).

Table S2. Expression of cytokeratin in the anal squamous epithelium.

Group			DSS		Control	
Sample number			Sample 1		Sample 1	
Parameter		Accession	Score	emPAI	Score	emPAI
Type I	CK10	A2A513	ND	ND	1482	1.48
	CK13	P08730	459	1.01	376	0.77
	CK42	Q6IFX2	ND	ND	131	0.2
Type II	CK1	P04104	ND	ND	1466	1.2
	CK4	P07744	ND	ND	356	0.46
	CK5	Q922U2	649	1.2	156	0.28
	CK6a	P50446	727	0.95	ND	ND
	CK8	P11679	ND	ND	140	0.18
	CK14	Q61781	670	1.11	ND	ND
	CK15	B1AQ77	449	0.85	ND	ND

Abbreviation: CK, Cytokeratin; emPAI, Exponentially modified protein abundance index; ND, Not detected.

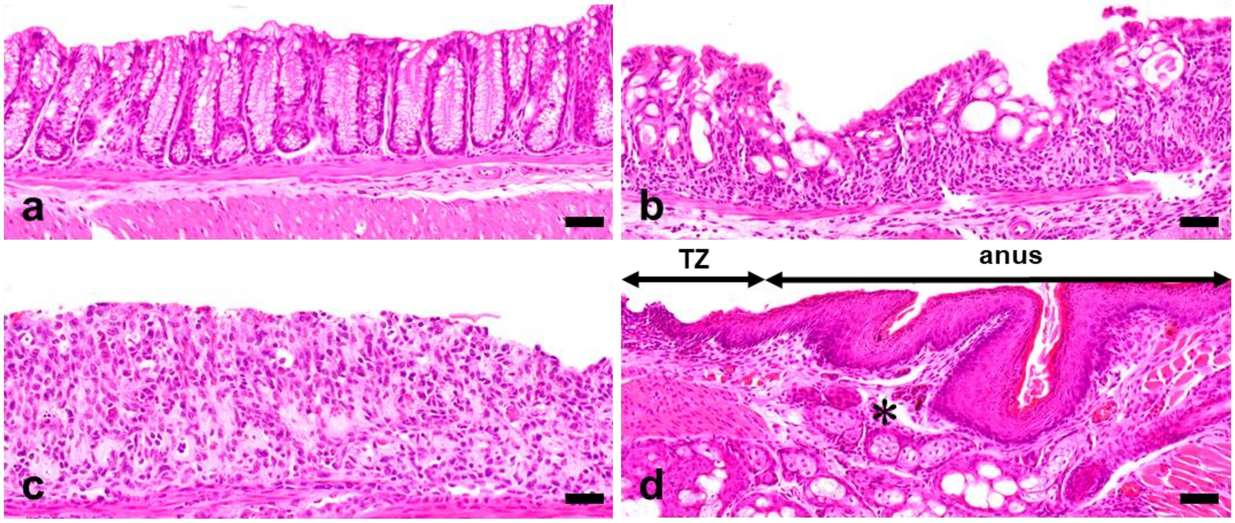

Figure S1. Representative images of the rectum and anus in the control and treated mice on Day 12. The mice were administered 5% DSS in drinking water for 6 days, followed by withdrawal of DSS for 6 days (Day 12). (a) The normal rectum in the control mouse. (b) Mild erosion in the proximal rectum in a DSS-treated mouse on Day 12. (c) Severe erosion in the distal rectum in a DSS-treated mouse on Day 12. (d) The TZ and perianal sebaceous glands (asterisk) in a DSS-treated mouse on Day 12. Hematoxylin and eosin stain. Bar=50 µm. DSS, dextran sodium sulfate; TZ, transitional zone.

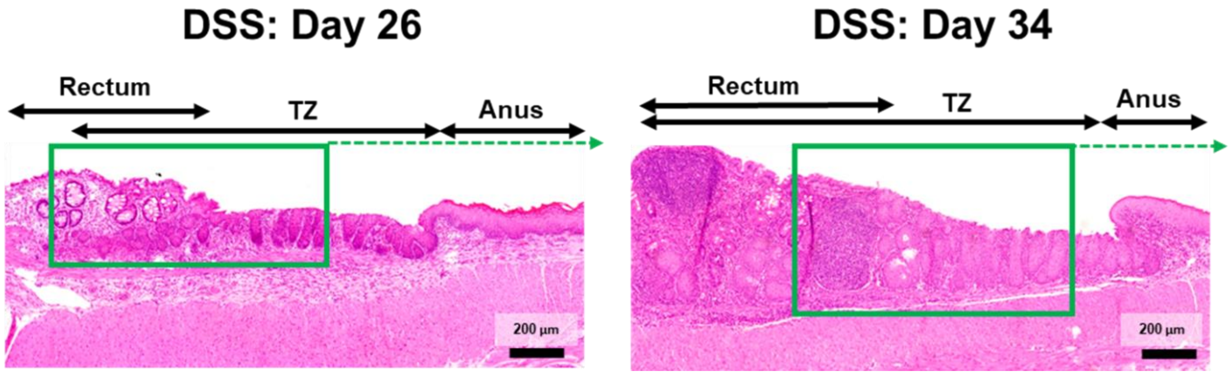

Figure S2. Low-magnification images of the TZ in the DSS-treated mice on Days 24 and 34. The green rectangles in the left and right images on Days 26 and 34 are shown in Fig. 1a. The abnormal TZ region, composed of nonkeratinizing squamous epithelial cells, extends toward the regenerating colonic mucosal epithelium, showing pathological remodeling. Hematoxylin and eosin stain. Bar=200 µm. DSS, dextran sodium sulfate; TZ, transitional zone.

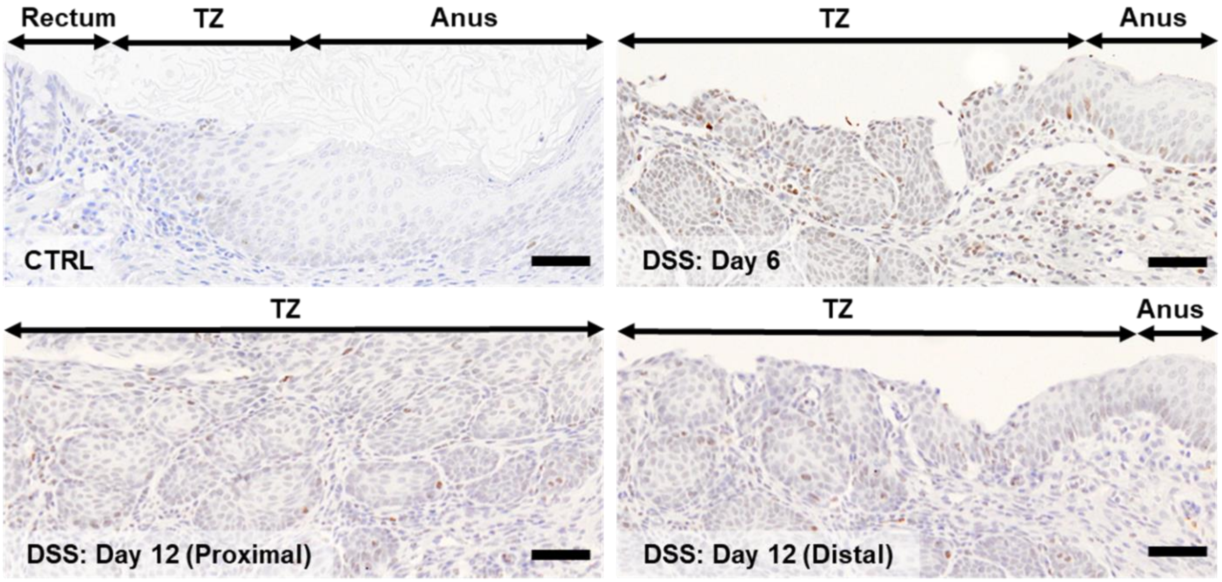

Figure S3. Representative images of nuclear expression of γ-H2AX at TZ in the control and treated mice on Day 12. The mice were administered 5% DSS in drinking water for 6 days (Day 6), followed by withdrawal of DSS for 6 days (Day 12). The TZ is observed between the rectum and anus. The weak positive reaction is observed in the TZ in the control and treated groups. The positive signals are visualized with 3,3'-diaminobenzidine as a chromogen (brown), followed by counterstaining with hematoxylin. Bar=50 μm. CTRL, control; DSS, dextran sodium sulfate; γ-H2AX, phosphorylation of histone H2AX at serine 139; TZ, transitional zone.

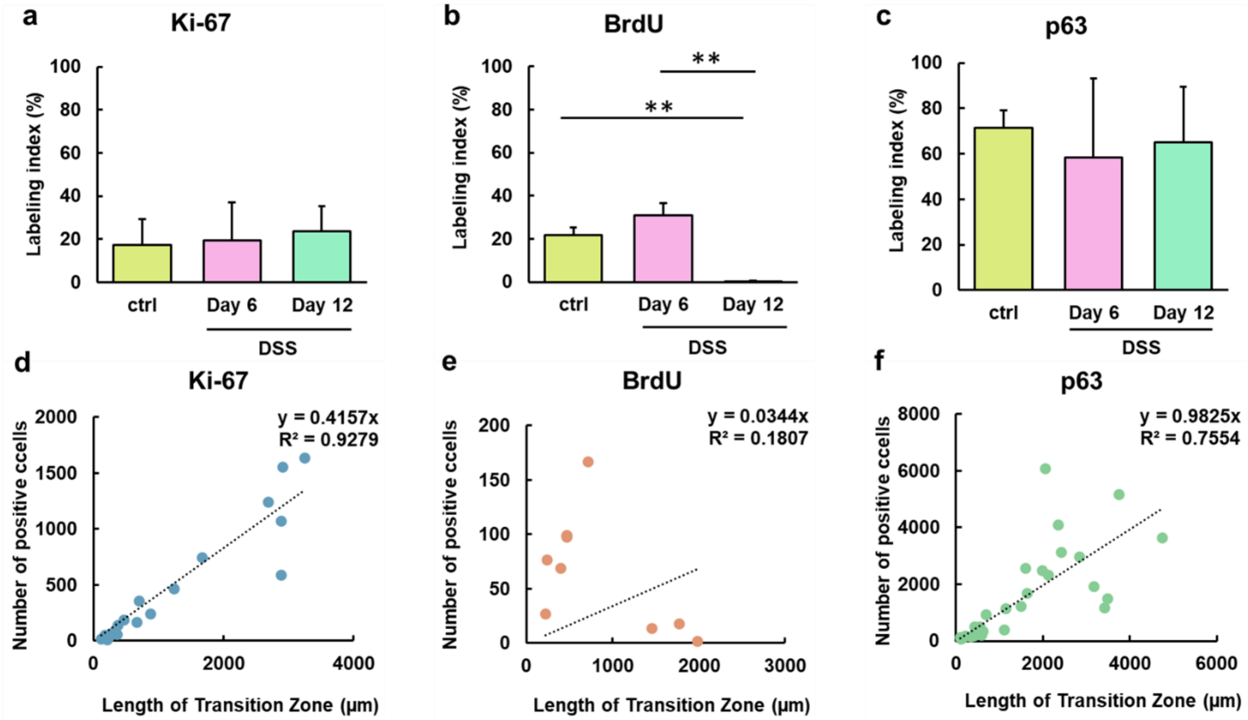

Figure S4. Labeling indices of Ki-67, BrdU, and p63, and correlation of these indices and the length of TZ. The labeling cells (see Fig. 2) were counted of Ki-67 (a), BrdU (b), and p63 (c) in the TZ. Scatter plots with correlation coefficient (R2) and linear function (y=ax) between the number of positive cells and the length of TZ in the labeling of Ki-67 (d), BrdU (e), and p63 (f). BrdU, 5-bromo-2'-deoxyuridine; DSS, dextran sodium sulfate; p63, tumor protein 63.

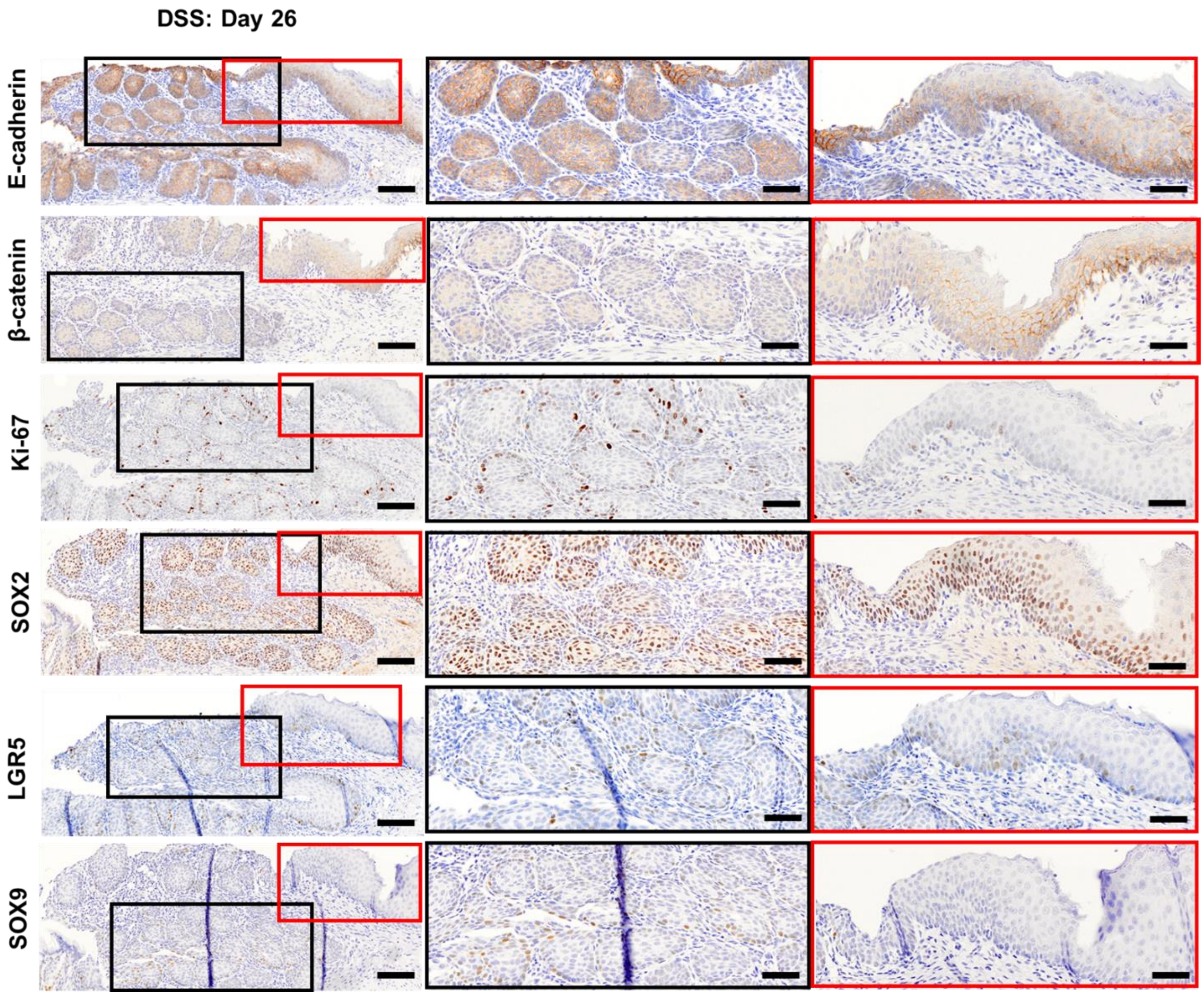

Figure S5. Immunohistochemistry of cell adhesion, cell proliferation, and stem cell markers in the rectum in the DSS-treated groups on Day 26. Representative images of E-cadherin, β-catenin, Ki-67, SOX2, LGR5, and SOX9 in the TZ in the treated mice administered 5% DSS in drinking water for 6 days, followed by withdrawal of DSS for 20 days (Day 26). The black and red rectangles on the left side of the images are shown at higher magnification on the center and right side, respectively. The positive signals are visualized with 3,3'-diaminobenzidine as a chromogen (brown), followed by counterstaining with hematoxylin. Bar = 100 μm in the left side images, and 50 μm in the center and right images. DSS, dextran sodium sulfate; LGR5, leucine-rich repeat-containing G-protein-coupled receptor 5; SOX2, sex-determining region on Y-box transcription factor 2; SOX9, Sex sex-determining region on Y-box transcription factor 9; TZ, transitional zone.

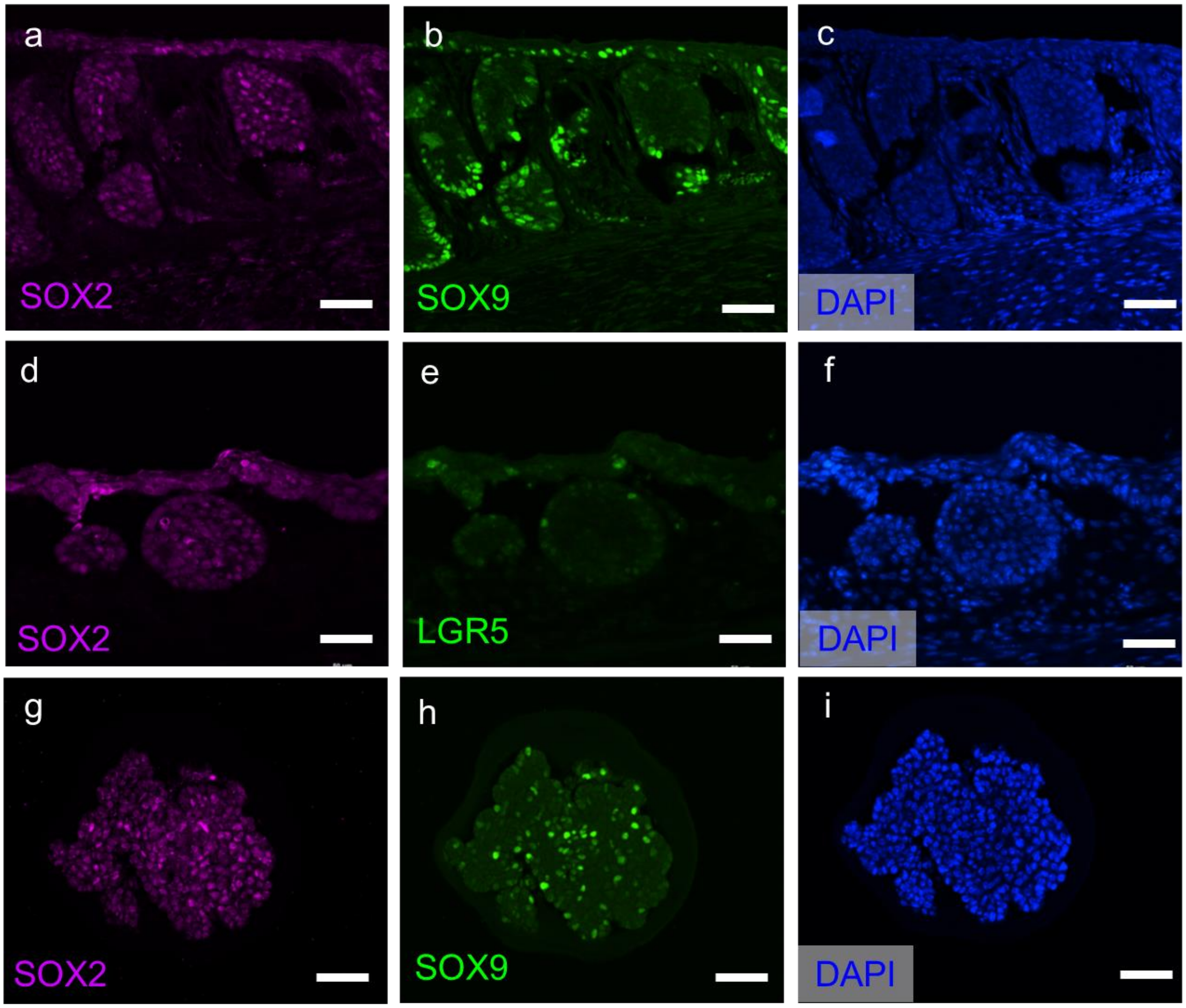

Figure S6. Non-merged images of Fig. 6. Representative images of TZ (a-f) and organoids of a non-keratinizing squamous cell (g-i) in the treated mouse administered 4% DSS in drinking water for 6 days, followed by withdrawal of DSS for 6 days (Day 12: a-f) and 21 days (Day 26; g-i). Immunofluorescence staining for SOX2 (Magenta: a, d, g), SOX9 (Green: b, h), LGR5 (Green: e), and DAPI (Blue: c, f, i). SOX2 is diffusely distributed in the TZ (a, d) and organoid (g). $SOX9^{high}$ and $SOX9^{low}$ cells are identified in the TZ (b) and organoid (h). LGR5 is weakly expressed in the TZ (e). Bar =50 µm. DSS, dextran sodium sulfate; LGR5, leucine-rich repeat-containing G-protein-coupled receptor 5; SOX2, sex-determining region on Y-box transcription factor 2; SOX9, Sex sex-determining region on Y-box transcription factor 9; TZ, transitional zone.

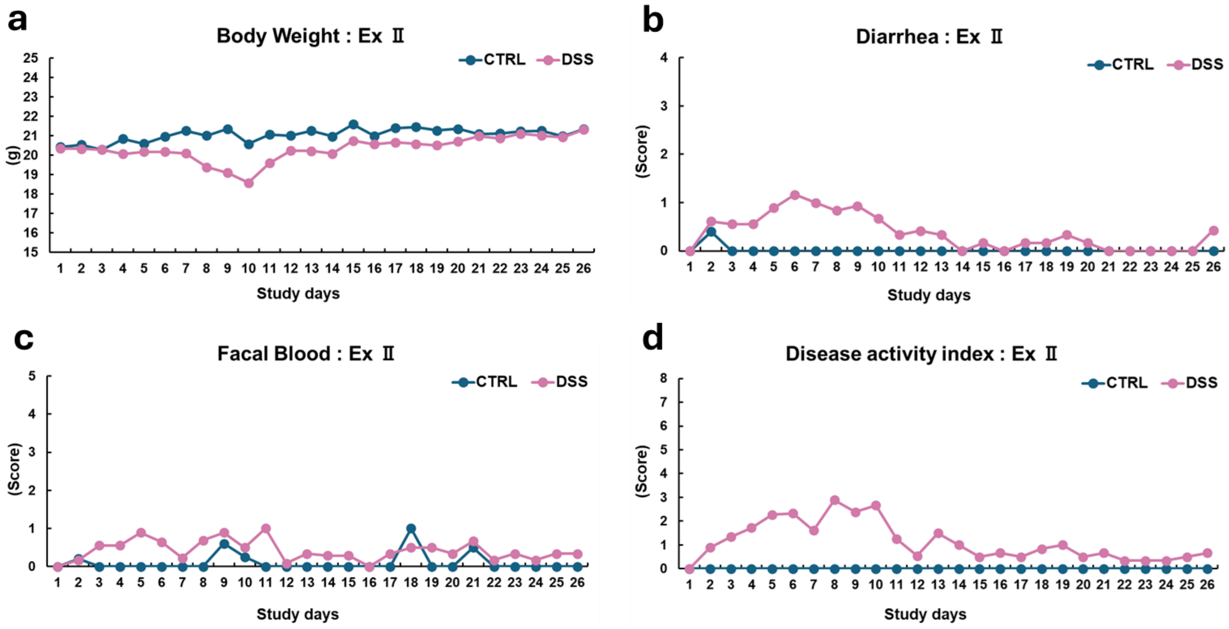

Figure S7. Disease activity index (DAI) in Experiment II. Mice were administered 4% DSS in drinking water for 6 days, followed by withdrawal of DSS for 6 (Day 12) or 20 days (Day 26). (A) Body weight (g). (B) Diarrhea (score). (C) Fecal blood (score). (D) DAI (score) is calculated by summing the scores for diarrhea, fecal blood, and body weight loss. The data represent the mean values for body weight, DAI, diarrhea scores, and fecal blood scores. N=4, control group (CTL); N=12, DSS-treated group. Ex: Experiment.

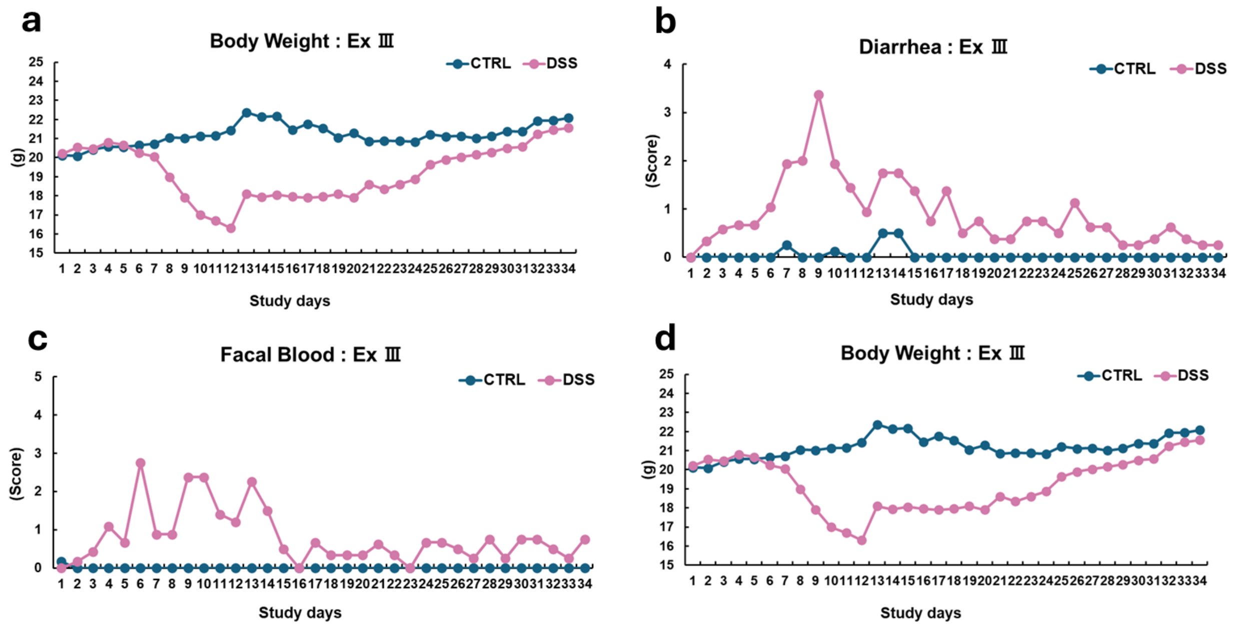

Figure S8. Disease activity index (DAI) in Experiment III. Mice were administered 4% DSS in drinking water for 6 days (Day 6), followed by withdrawal of DSS for 6 (Day 12) or 28 days (Day 34). (A) Body weight (g). (B) Diarrhea (score). (C) Fecal blood (score). (D) DAI (score) is calculated by summing the scores for diarrhea, fecal blood, and body weight loss. The data represent the mean values for body weight, DAI, diarrhea scores, and fecal blood scores. N=6, control group (CTL); N=12, DSS-treated group. Ex: Experiment.

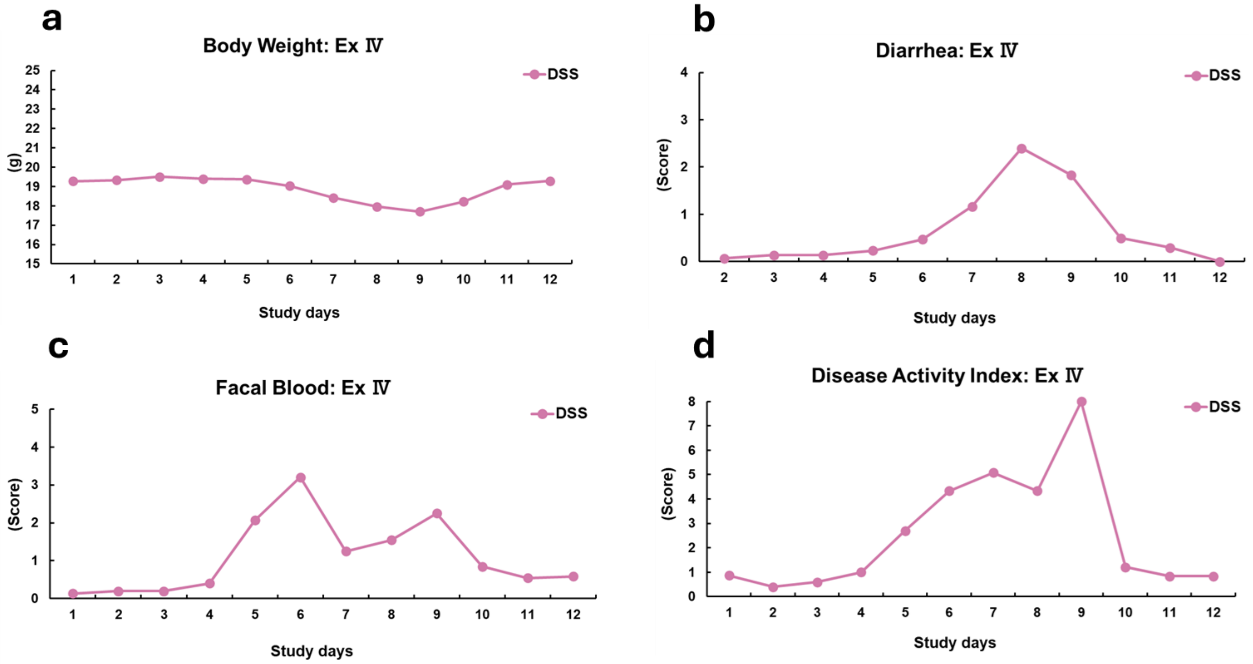

Figure S9. Disease activity index (DAI) in Experiment IV. Mice were administered 4% DSS in drinking water for 6 days (Day 6), followed by withdrawal of DSS for 6 (Day 12). (A) Body weight (g). (B) Diarrhea (score). (C) Fecal blood (score). (D) DAI (score) is calculated by summing the scores for diarrhea, fecal blood, and body weight loss. The data represent the mean values for body weight, DAI, diarrhea scores, and fecal blood scores. N=6, control group (CTL); N=12, DSS-treated group. Ex: Experiment.

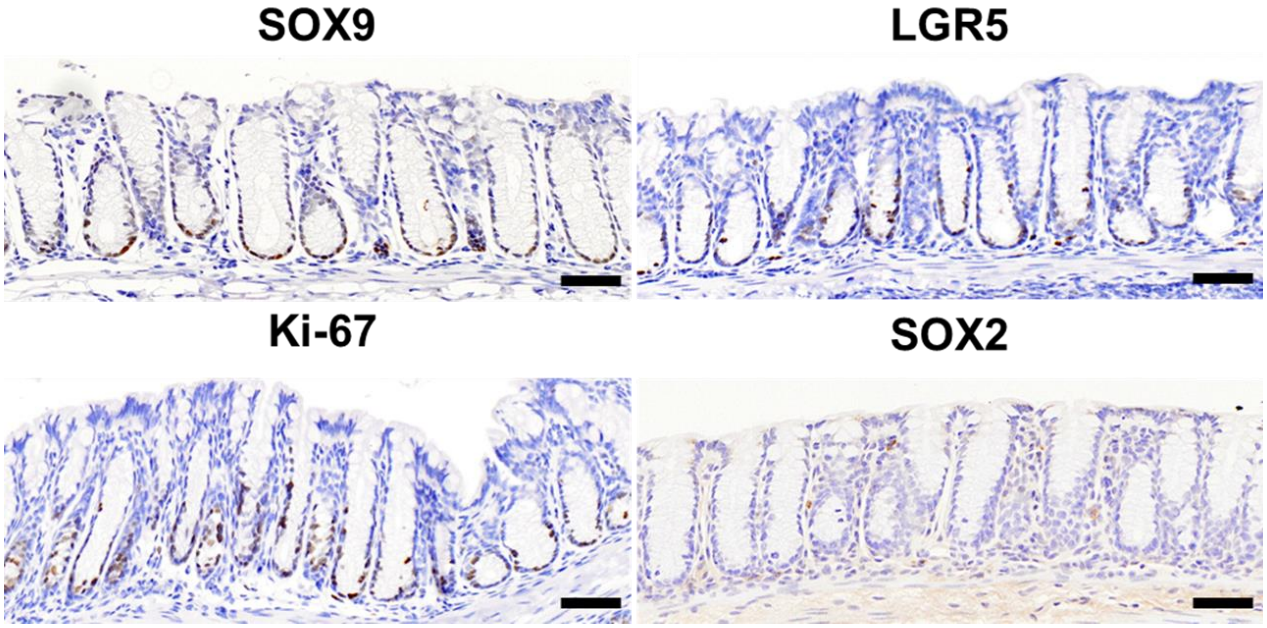

Figure S10. Immunohistochemistry of stem cell and cell proliferation markers in the rectum in the control group. Representative images of SOX9, LGR5, Ki-67, and SOX2 in the crypts. The depth of crypts expresses SOX9, LGR5, and Ki-67, but not SOX2. The positive signals are visualized with 3,3'-diaminobenzidine as a chromogen (brown), followed by counterstaining with hematoxylin. Bar=50 μm. DSS, dextran sodium sulfate; LGR5, leucine-rich repeat-containing G-protein-coupled receptor 5; SOX2, sex-determining region on Y-box transcription factor 2; SOX9, Sex-determining region on Y-box transcription factor 9; TZ, transitional zone

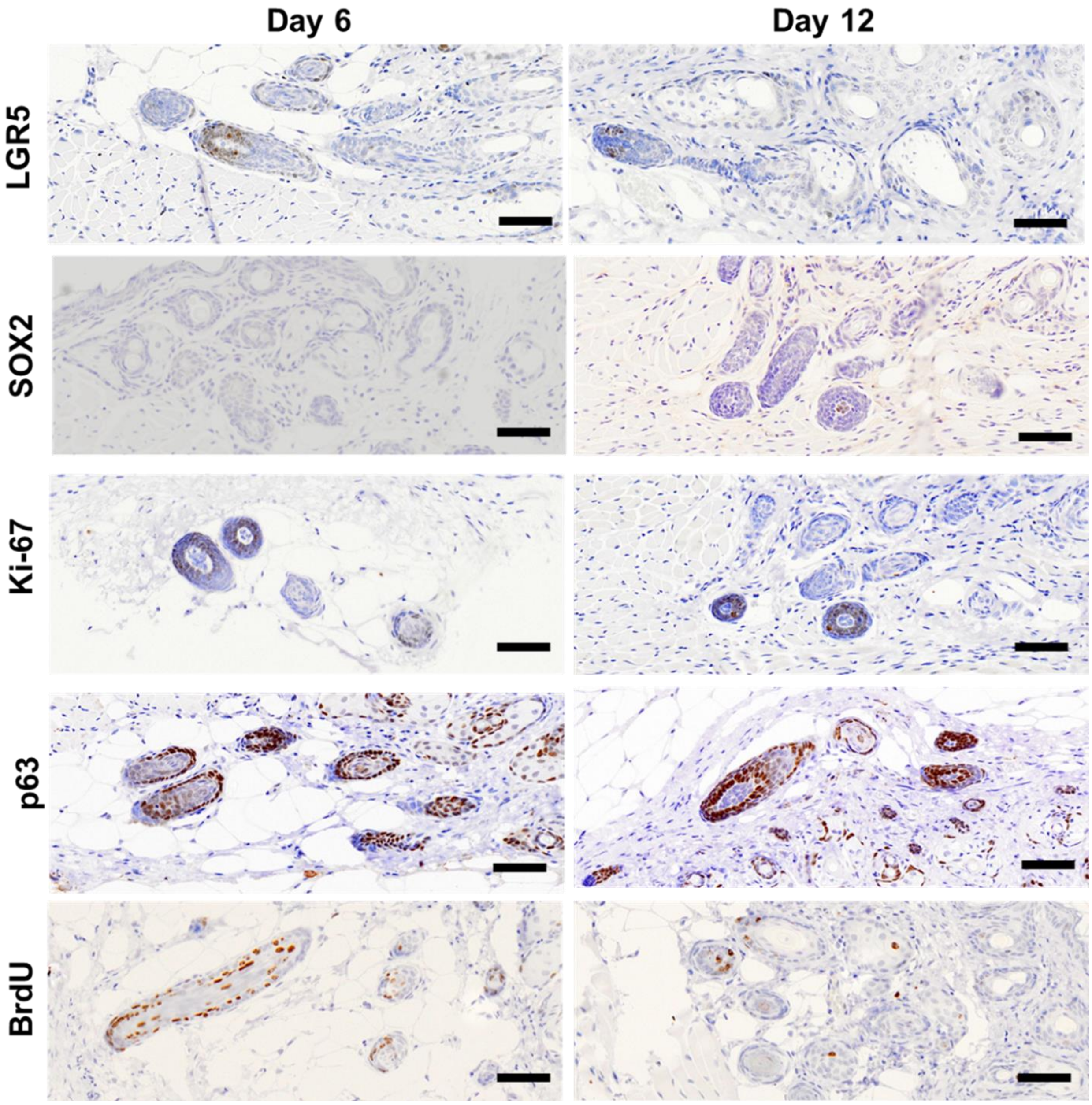

Figure S11. Immunohistochemistry of stem cell and cell proliferation markers in the anal skin in the DSS-treated group on Day 12. Representative images of positive signals of Lgr5, Ki-67. p63 and BrdU in the follicles in the treated mice administered 5% DSS in drinking water for 6 days (Day 6), followed by withdrawal of DSS for 6 days (Day 12). The positive signals are visualized with 3,3'-diaminobenzidine as a chromogen (brown), followed by counterstaining with hematoxylin. Bar=50 µm. BrdU; 5-bromo-2′-deoxyuridine; DSS, dextran sodium sulfate; LGR5, leucine-rich repeat-containing G-protein-coupled receptor 5; p63, tumor protein 63; SOX2, sex-determining region on Y-box transcription factor 2; SOX9, sex-determining region on Y-box transcription factor 9; TZ, transitional zone.

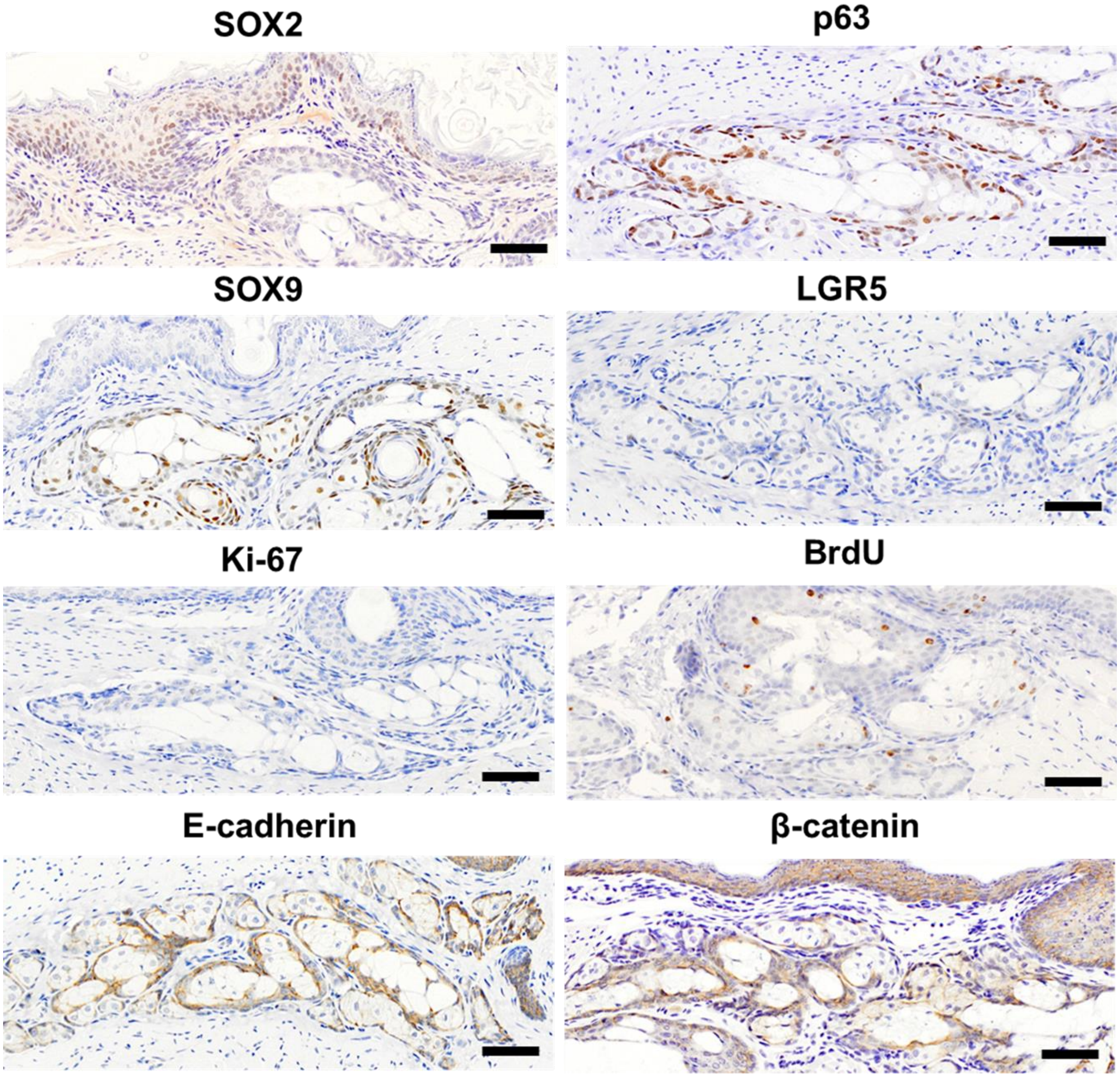

Figure S12. Immunohistochemistry of stem cell, cell proliferation, and cell adhesion markers in the anus in the DSS-treated group. Representative images of SOX2, p63, SOX9, LGR5, Ki-67, BrdU, E-cadherin, and β-catenin in the sebaceous glands under the anal epithelium in the treated mice administered 5% DSS in drinking water for 6 days, followed by withdrawal of DSS for 6 days (Day 12). The sebaceous glands highly express p63, SOX9, E-cadherin, and β-catenin, and are labelled with BrdU, whereas those less express Lgr5 and Ki-67. The positive signals are visualized with 3,3’-diaminobenzidine as a chromogen (brown), followed by counterstaining with hematoxylin. Bar=50 μm. BrdU; 5-bromo-2′-deoxyuridine; DSS, dextran sodium sulfate; LGR5, leucine-rich repeat-containing G-protein-coupled receptor 5; p63, tumor protein 63; SOX2, sex-determining region on Y-box transcription factor 2; SOX9, sex-determining region on Y-box transcription factor 9; TZ, transitional zone.

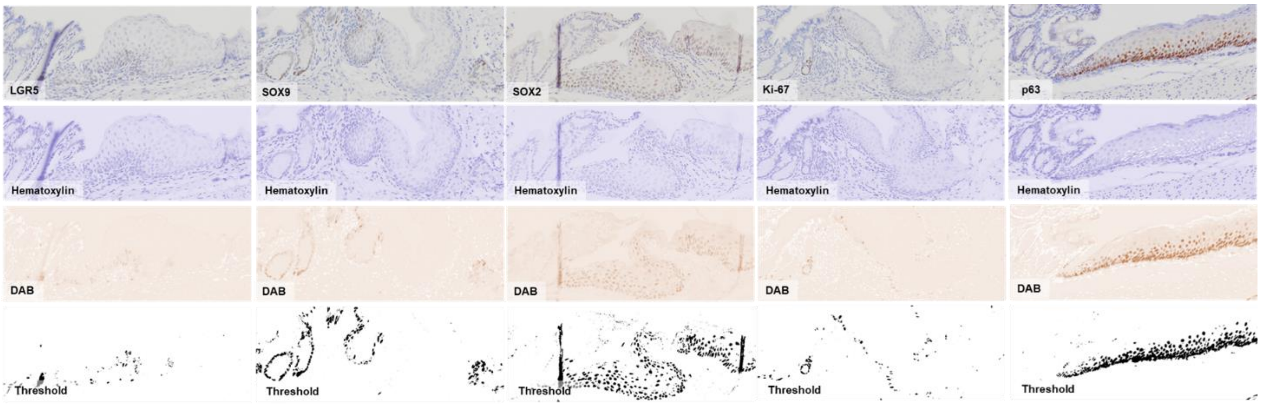

Figure S13. Detection of positive signals in immunohistochemistry in the TZ in the DSS-treated group using Fiji (https://imagej.net/software/fiji/downloads). Representative images of immunostaining of LGR5, SOX9, SOX2, Ki-67, and p63 in the first line. Fiji revealed the images of background (hematoxylin) stain and positive signals (DAB) of each target protein in the second and third layers, respectively. Each of the images was extracted for the target color using Colour Deconvolution, which, in turn the upper and lower limits of threshold at positive cells were set based on the control group. The lower limit of LGR5 was 0, and the upper limit was 193, SOX9 was 0 and 200, SOX2 was 0 and 188, Ki-67 was 0 and 197, and p63 was 0 and 180. Finally, binalyzed positive signals are shown in the fourth layer. Bar=50 μm. DAB, 3,3'-diaminobenzidine; DSS, dextran sodium sulfate; LGR5, leucine-rich repeat-containing G-protein-coupled receptor 5; p63, tumor protein 63; SOX2, sex-determining region on Y-box transcription factor 2; SOX9, sex-determining region on Y-box transcription factor 9; TZ, transitional zone.

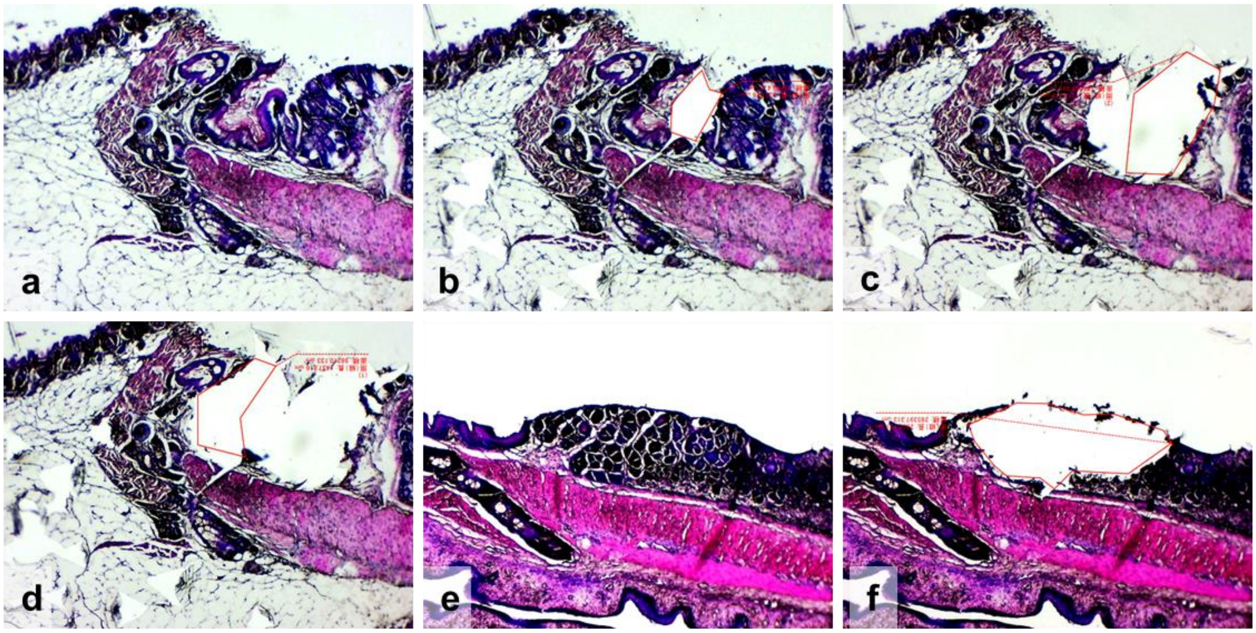

Figure S14. Microdissection of the TZ in the DSS-treated mice for LC-MS/MS. Representative images of anorectum in the control (a-d) and treated mice (e and f) administered 5% DSS in drinking water for 6 days, followed by withdrawal of DSS for 6 days (Day 12). (a) Intact images in the control. The anus is seen the left side, and the rectum is seen in the right side. (b) The blank space is the region of the excised TZ (compare with the image (a)). (c) The blank space is the region of the excised TZ and rectum (compare with the images (a) and (b)). (d) The blank space is the region of the excised anus, TZ and rectum (compare with the images (a-c)). (e) Pseudo-carcinomatous hyperplasia in a DSS-treated mouse on Day 12. (f) The blank space is the region of the excised pseudo-carcinomatous hyperplasia (compare with the images (e)). The tissues at 7 um in thickness were stained with hematoxylin and eosin stain. DSS, dextran sodium sulfate; LC-MS/MS, liquid chromatograph-tandem mass spectrometry; TZ, transitional zone.
